# Supplementary material for: Harnessing the benefits of diversity to address socio-environmental governance challenges
Source: PLoS One. 2022 Aug 10;17(8):e0263399. doi: 10.1371/journal.pone.0263399 (PMC9365146; doi:10.1371/journal.pone.0263399)
Supplement: S1 File — (PDF) [file pone.0263399.s001.pdf]

Supplementary Information for Harnessing the benefits of diversity to address socio-environmental governance challenges.

Jacopo A. Baggio[1,2*◑], Jacob Freeman[3,4,◑], Thomas R. Coyle[5], John M. Anderies[6,7]

1 School of Politics, Security, and International Affairs, University of Central Florida, Orlando, USA
2 National Center for Integrated Coastal Research, University of Central Florida, Orlando, USA
3 Anthropology Program, Utah State University, Logan, UT USA
4 The Ecology Center, Utah State University, Logan, UT USA
5 Department of Psychology, University of Texas at San Antonio, San Antonio, TX USA
6 School of Sustainability, Arizona State University, Tempe, AZ USA
7 School of Human Evolution and Social Change, Arizona State University, Tempe, AZ USA

◑These authors contributed equally to this work.

Supplementary Figures

Problem complexity as well as g and ToM have a clear effect on the ability of groups to engage in collective action apt to find appropriate solutions as shown in Fig. S1. On average, solutions are harder to find for increased levels of social and/or ecological complexity(Fig. S1a), and easier the higher the levels of g and ToM (Fig. S1b)

(a) **(b)**

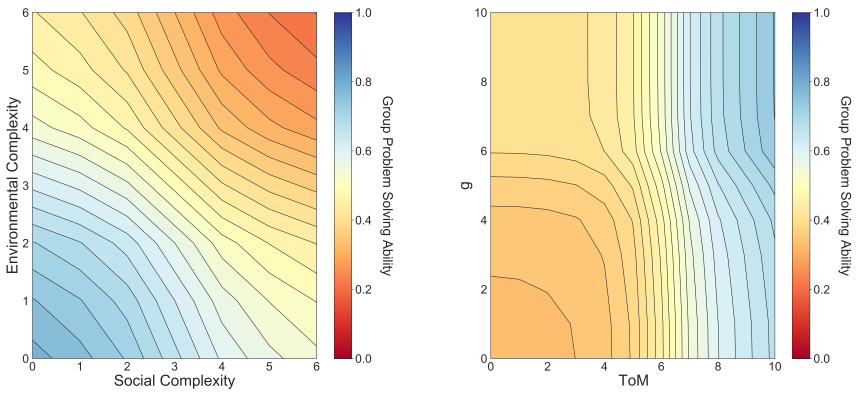

.

Fig. S1. (a) Effect of social and environmental complexity on the ability of groups to find solutions to problems, independent on levels of g and ToM; (b) Effect of g and ToM on finding problem solutions, independent of problem complexity.

Further, we can assess how g, ToM, social and environmental complexity affect groups problem solving ability for different levels of α and β.

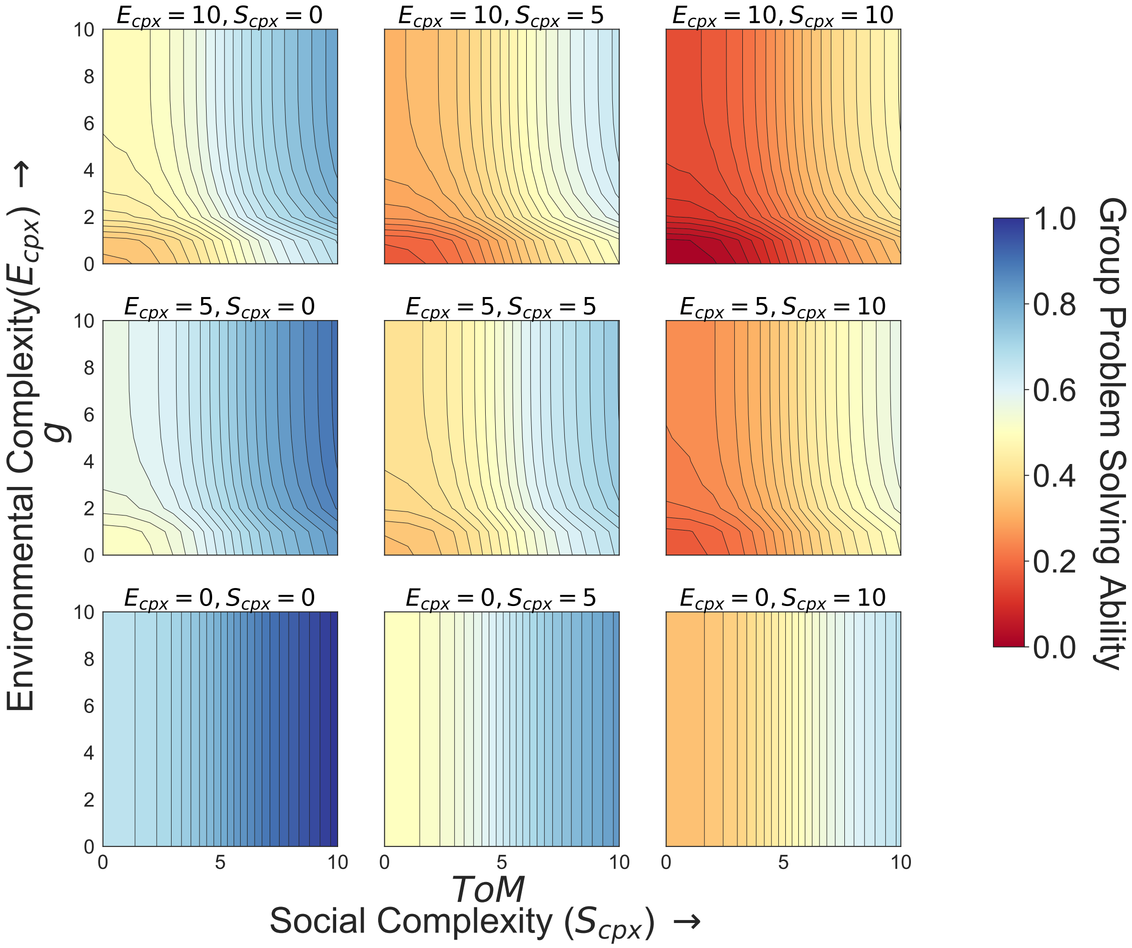

Fig. S2. Effect of g and ToM for different levels of social and environmental complexity for $\alpha = \beta = 1$.

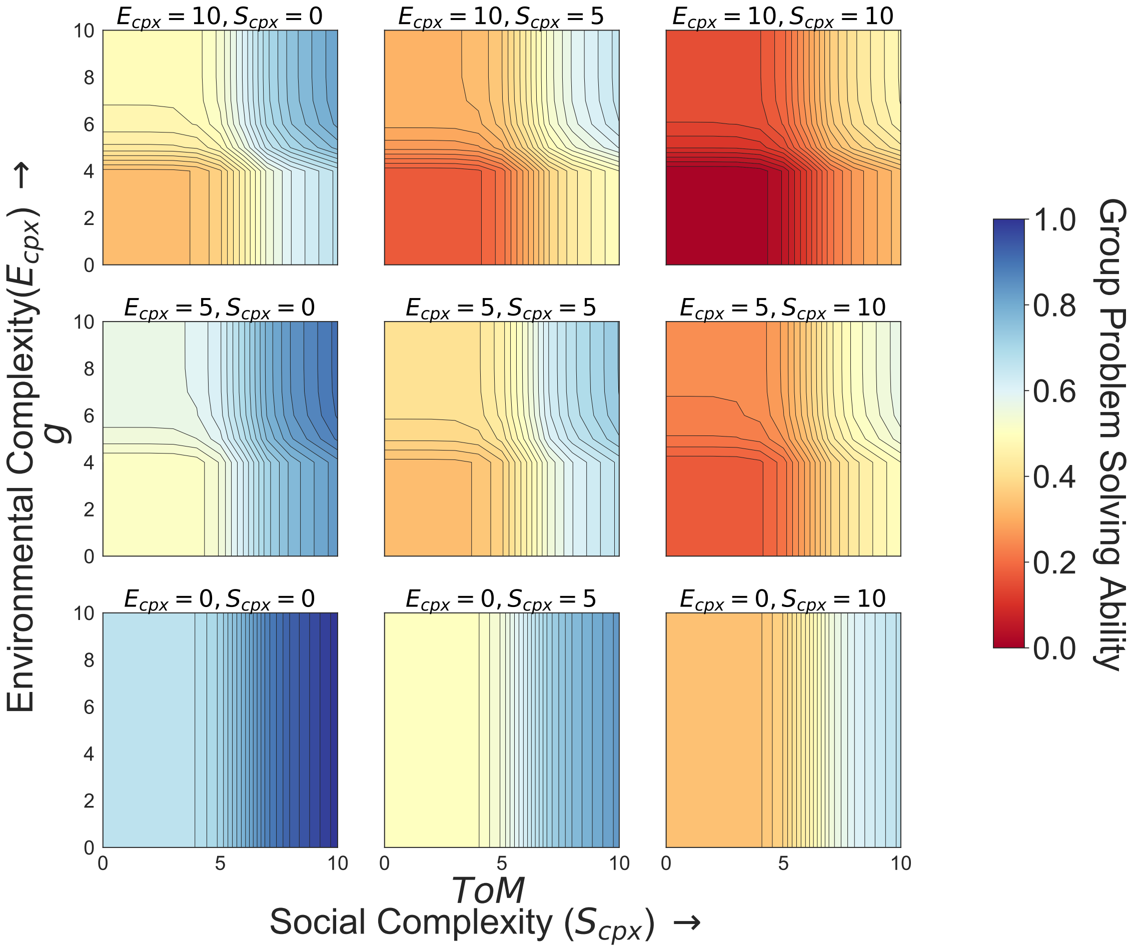

Fig. S3. Effect of g and ToM for different levels of social and environmental complexity for $\alpha = \beta = 5$.

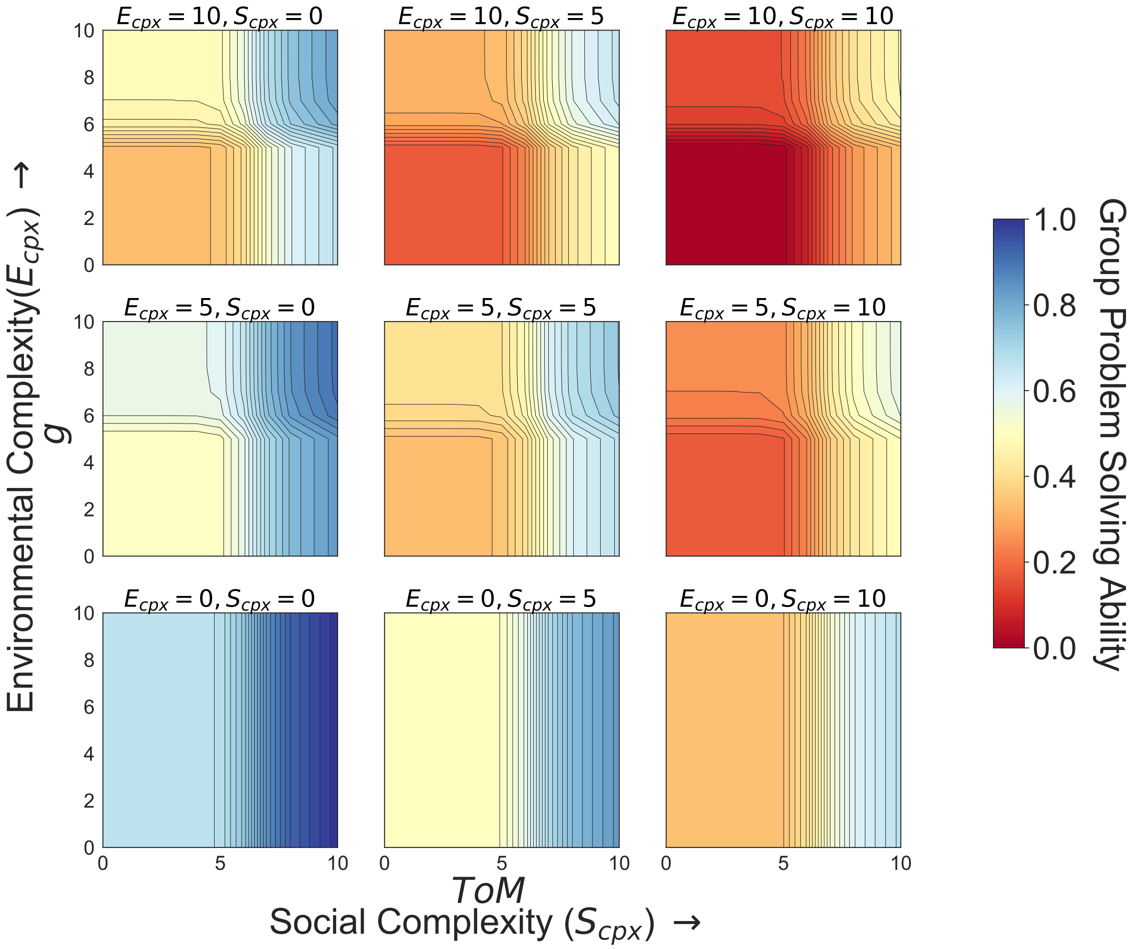

Fig. S4. Effect of g and ToM for different levels of social and environmental complexity for $\alpha = \beta = 10$.

Similarity of g, ToM and problem solution across scales

We assess the similarity of the distribution of cognitive abilities at the different levels examined. We assess this visually by fitting a kernel density estimation.

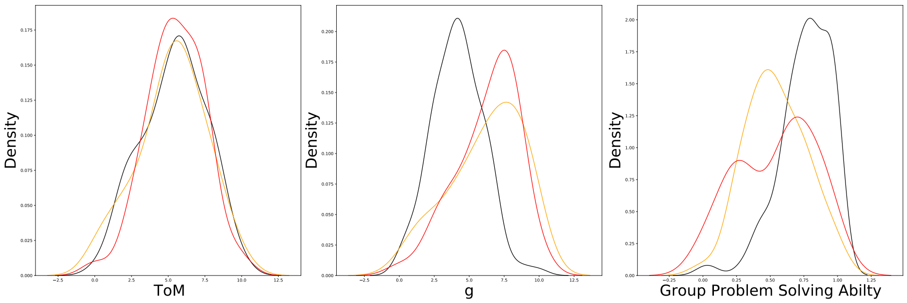

Fig. S5. Distribution of g, ToM and collective action ability at multiple levels (Small-groups (black), U.S. States (orange) and Countries (red)

ToM has the same distribution from small-groups to countries (Fig. S5). ToM, is basically "scale invariant", at least, with respect to the countries examined here. g distribution is similar at the US State and Country level, but different with respect to small groups.Group problem solving ability is different for all levels Fig. S5.

Hence, ToM seem to follow similar distribution independent on whether we are looking at groups, U.S. States or Countries. On the other hand, g is similarly distributed when it comes to U.S. States and Countries, and GPSA is differently distributed at all three levels taken into account.
