# Supplementary material for: Harnessing the benefits of diversity to address socio-environmental governance challenges
Source: PLoS One. 2022 Aug 10;17(8):e0263399. doi: 10.1371/journal.pone.0263399 (PMC9365146; doi:10.1371/journal.pone.0263399)
Supplement: Attachment — Submitted filename: Response_Minors.docx [file pone.0263399.s004.docx]

Once again, we would really like to thank reviewers and the editor for the suggestions and comments that have really strengthen this manuscript. Please see our responses to the remaining queries below.

**Reviewer #2:**

1) Rdiv is such an important intervening variable that it has a dedicated section in the model description. On page 3, “cognitive representational diversity” is also listed as one of three important concepts, together with cognitive tools and cognitive abilities. Despite this, it is now difficult to relate Rdiv to the other concepts and the relationship to Scpx is also not very clear. For instance, is “increasing the stakeholder diversity” (p. 4) about increasing Scpx or about Rdiv, or about both but in different contexts (entire community vs. specific action situation)? Perhaps Rdiv might be added to Fig. 1 somewhere (e.g. in the “Action Situation” box?) and a few more conceptual clarifications can be made in text.

**Response:** Thank you for this comment. We were not as clear as we could have been. Cognitive representational diversity actually has two dimensions that we have now clearly parsed. The first is mechanical--the different ways that individuals represent the mechanics of a system--and the second is values based--the different values and objectives that individuals bring to an action situation. We have now made this clear by revising the introduction, the text on page 3 where we define cognitive representational diversity, and in the text on page 4. It should now be clear that as social complexity increases, both dimensions of cognitive representational diversity increase. So, Scpx is external to the Action Situation. As scpx goes up, so does Rdiv. Rdiv includes both the mechanical and the value dimensions of cognitive representational diversity; this is why Rdiv is an important term in both equations 1 and 3. As Rdiv increases, mechanical and value representations of a system also increase; that is, these dimensions positively covary. This is why we argue that increasing Rdiv supercharges learning, as long as a process is in place for all to share their views, but increases in Rdiv may lead to conflict as values and objective clash even in cases where all can share their views. In essence ToM and $\beta$ moderate the positive correlation of mechanical and value cognitive representational diversity (equations 3 and 4). More ToM and lower $\beta$, in effect, weaken the correlation between mechanical cognitive div. and value/objective cognitive div. We have added representational diversity and gaps within the action situation to clarify further the argument made here.

2) On page 5 it is stated: “greater socio-environmental complexity requires that groups increase their representational diversity to supercharge learning…” – and this is used as an assumption in the model. Later, a similar statement appears as a result (result 6 on p.14). A clarification or a refutation of circularity of argument is needed.

**Response:** NOTE: We don't present ``results on page 6, so I am assuming the reviewer means the ``model results" section.

We have now clarified that this is an assumption. On page 5 we present insights of visually representing the interaction between the formal model's variables and parameters. The `results' here are really modeled results or clarifications of our hypotheses based on how our assumptions lead to outcomes in the formal model space. We have attempted to make this clear in the model results section.

3) Page 6, where alpha is introduced: “The parameter alpha represents the cognitive tools affecting…” – perhaps good to mention already here that high value is actually “high restriction on access to tools”, because based on the definition one would initially assume that high value = more cognitive tools = better.

**Response:** The reviewer is correct, we have amended the text. We have added the same language when we introduce the parameter beta.

4) Consider checking Fig 4, Fig 5 and those in the supplementary information for readability in black/white.

**Response:** Indeed, the only way to do this would be to have a monochromatic scale, given that Plos one is an online only journal, we are unsure whether this would allow for a clearer interpretation of the figures compared to the coolwarm scale chosen.

5) Consider adding Rdiv, Rhdg, wdiv and wgap to Table 1.

**Response:** This could be a great idea, however, table 1 represent parameters and variables of the model, while the four aforementioned model elements are a function of these parameters in the work presented, hence, it may create confusion to add them in the table, as they would entail rewriting the functions in the behavioral and cultural/ecological inheritance loops.

6) A few typos and small errors remain, e.g. my previous comment about “hypotheses” – the singular form is now used instead of plural (p.17).

**Response:** Corrected, we hope to have addressed all remaining typos.
